# Multiple intravenous injections of allogeneic equine mesenchymal stem cells do not induce a systemic inflammatory response but do alter lymphocyte subsets in healthy horses

**DOI:** 10.1186/s13287-015-0050-0

**Published:** 2015-04-15

**Authors:** Amir Kol, Joshua A Wood, Danielle D Carrade Holt, Jessica A Gillette, Laurie K Bohannon-Worsley, Sarah M Puchalski, Naomi J Walker, Kaitlin C Clark, Johanna L Watson, Dori L Borjesson

**Affiliations:** Department of Pathology, Microbiology and Immunology, School of Veterinary Medicine, University of California, One Shields Avenue, Davis, CA 95616 USA; Department of Surgical and Radiological Sciences, School of Veterinary Medicine, University of California, Davis, CA 95616 USA; Department of Medicine and Epidemiology, School of Veterinary Medicine, University of California, Davis, CA 95616 USA

## Abstract

**Introduction:**

Intravenous (IV) injection of mesenchymal stem cells (MSCs) is used to treat systemic human diseases and disorders but is not routinely used in equine therapy. In horses, MSCs are isolated primarily from adipose tissue (AT) or bone marrow (BM) and used for treatment of orthopedic injuries through one or more local injections. The objective of this study was to determine the safety and lymphocyte response to multiple allogeneic IV injections of either AT-derived MSCs (AT-MSCs) or BM-derived MSCs (BM-MSCs) to healthy horses.

**Methods:**

We injected three doses of 25 × 10^6^ allogeneic MSCs from either AT or BM (a total of 75 × 10^6^ MSCs per horse) into five and five, respectively, healthy horses. Horses were followed up for 35 days after the first MSC infusion. We evaluated host inflammatory and immune response, including total leukocyte numbers, serum cytokine concentration, and splenic lymphocyte subsets.

**Results:**

Repeated injection of allogeneic AT-MSCs or BM-MSCs did not elicit any clinical adverse effects. Repeated BM-MSC injection resulted in increased blood CD8^+^ T-cell numbers. Multiple BM-MSC injections also increased splenic regulatory T cell numbers compared with AT-MSC-injected horses but not controls.

**Conclusions:**

These data demonstrate that multiple IV injections of allogeneic MSCs are well tolerated by healthy horses. No clinical signs or clinico-pathologic measurements of organ toxicity or systemic inflammatory response were recorded. Increased numbers of circulating CD8^+^ T cells after multiple IV injections of allogeneic BM-MSCs may indicate a mild allo-antigen-directed cytotoxic response. Safety and efficacy of allogeneic MSC IV infusions in sick horses remain to be determined.

## Introduction

Mesenchymal stem cells (MSCs) have been isolated from humans and most veterinary and laboratory animal species, including horses [1,2]. In horses, MSCs have primarily been isolated and characterized from adipose tissue (AT), bone marrow (BM), umbilical cord blood, and umbilical cord tissue [3]. The ideal MSC dose for any medical application has not been determined. Autologous and allogeneic doses of 10 to 80 × 10^6^ equine MSCs are currently used in clinical applications for tissue regeneration and repair as well as immunomodulation [3,4].

Allogeneic MSCs offer considerable advantages over autologous MSCs as they do not require patient-specific tissue harvest, they are available for immediate application, and cell batches can be well characterized, providing a consistent source of multiple cell doses [5]. In addition to MSC tissue source, the route of MSC administration is an important consideration for therapeutic applications. Equine autologous and allogeneic MSCs have been safely administered by regional and local injection routes [5-7]. In human clinical trials, intravenous (IV) injection offers a readily accessible injection route for systemic MSC administration and permits therapeutic application to patients with systemic inflammatory or immune-mediated diseases (cardiovascular disease, respiratory disease, and gastrointestinal disease) [8-10]. Although the IV administration of MSCs to treat horses has not yet been evaluated, IV injection may be increasingly used in equine medicine as we move toward cell-based therapy for systemic inflammatory diseases such as respiratory or gastrointestinal diseases. However, MSCs may be recognized as foreign by the immune system and this can result in a systemic inflammatory response directed at the cells or, at the very least, immune destruction of these cells with a resultant decrease in MSC life span and efficacy. A safety study demonstrated that a single dosage of 0.2 to 1 × 10^6^ IV allogeneic MSCs in 291 horses was not associated with any clinical adverse effects [11]. This study demonstrated the safety of IV allogeneic MSC administration in a large cohort of healthy horses. In our study, we administered three MSC doses that are 25- to 125-fold higher than what was previously reported. Moreover, only a single tissue source (peripheral blood) of MSCs was used and only clinical outcomes were recorded [11].

Multiple MSC injections may be therapeutically advantageous for orthopedic and immunomodulatory conditions [4]. The current standard of care in human patients often involves multiple injections, frequently by different routes of administration (for example, a local injection followed by a regional injection). Safety after multiple local MSC injections in horses has largely been demonstrated [5,7]; however, there is limited detailed information on the safety of repeated IV allogeneic MSC injections in horses [5,11].

MSCs modulate anti-inflammatory cytokine secretion and leukocyte phenotype ratios both *in vitro* and *in vivo. In vitro*, MSCs inhibit T- and B-cell proliferation in both humans and horses [1,12]. In humans, MSCs secrete T cell-modulating factors, including prostaglandin E_2_ (PGE_2_), and control regulatory T (Treg) cell function [13,14]. Equine MSCs inhibit inflammatory mediator production—that is, interferon-gamma (IFN-γ) and tumor necrosis factor-alpha (TNF-α)—while upregulating the production of T cell-modulating and anti-inflammatory cell mediators (that is, transforming growth factor-beta (TGF-β) and PGE_2_) [3,15]. Recently, equine MCSs were reported to have variable major histocompatibility complex II (MHC-II) expression, which was correlated with induction of T-cell proliferation *in vitro* [16]. *In vivo*, MSCs modulate CD4^+^ and CD8^+^ T-cell numbers [10,17]. It is unknown whether systemically administered equine MSCs alter inflammatory cytokine levels, leukocyte numbers, or Treg cell numbers *in vivo*.

The objective of this study was to test the safety of multiple, allogeneic IV MSC doses in horses. We hypothesized that repeated IV MSC administration from either AT or BM would not elicit a systemic inflammatory immune response. We further hypothesized that IV MSC administration would result in increased Treg cell numbers. To test these hypotheses, we measured blood and splenic leukocyte numbers and subsets and systemic serum cytokine concentrations over the course of three MSC injections. We found that multiple IV injections of allogeneic MSCs are well tolerated in healthy horses. Allogeneic BM-derived MSCs (BM-MSCs) may induce a mild immune response that is characterized by an increased percentage of CD8^+^ T cells in the peripheral blood.

## Methods

### Study design

This study was conducted in accordance with an approved institutional animal care and use committee protocol. In total, 12 horses were enrolled in the study: 10 treatment and two controls. All horses were healthy adults (5 to 14 years old, five females, seven geldings, one quarter horse, 11 thoroughbreds) housed at the UC Davis Center for Equine Health. Low-passage (P3-P5) BM- and AT-derived MSCs from five and five horses, respectively, were obtained from the UC Davis William R. Pritchard Veterinary Medical Teaching Hospital (VMTH), Regenerative Medicine Laboratory. These MSCs were originally expanded for autologous patient treatment. Excess cells not used for treatment were donated for research purposes, with written owner consent. None of the MSC donor horses was enrolled in the study. MSCs were cultured and formulated for IV administration exactly as previously described [5,7]. Briefly, on the morning of the treatment, MSCs were detached with 0.05% Trypsin-EDTA solution (Gibco, Grand Island, NY, USA), neutralized, and washed twice with phosphate buffer solution. Each horse was injected with MSCs from the same allogeneic donor throughout the course of the study. Splenic aspirates and peripheral blood were collected from four additional healthy horses (5 to 14 years old, four females, three thoroughbreds, one quarter horse) to obtain reference ranges for all analytes where ranges were not previously published. To minimize the number of animals needed, all data from day 0 samples (collected prior to any MSC administration) were included in the reference ranges. All of the horses served as their own controls and baseline data served as control for any statistical analysis. We compared the data from the two control horses with the baseline data to confirm that there was no sham injection/placebo effect.

### Mesenchymal stem cell doses

AT- or BM-MSCs (25 × 10^6^ per injection) were administered on days 0, 14, and 28 (a total of 75 × 10^6^ MSCs per horse) in 3 mL of sterile saline (Baxter, Deerfield, IL, USA) by IV injection at a rate of 2 × 10^6^ cells per minute. This administration rate was selected on the basis of previous experience in feline MSC transfusion at the UC Davis VMTH. We have not observed any transfusion reactions at this rate of cell delivery.

### Clinical study and tissue collection

Temperature, pulse, and respiration data were recorded prior to sedation. Horses were sedated with detomidine hydrochloride (0.01 mg/kg; Pfizer Animal Health, Exton, PA, USA)/butorphanol tartrate (0.01 mg/kg; Fort Dodge Animal Health, Fort Dodge, IA, USA). Blood (60 mL) was collected prior to each MSC injection (days 0, 14, and 28) and on day 35 from all horses. Temperature, pulse, and respiration rate were formally recorded prior to MSC therapy, and horses were monitored throughout the time of administration and for up to 2 hours after administration (watching for respiratory stress and so on). Horses were further checked daily to monitor any adverse effects. Blood was submitted to the hematology laboratory at the VMTH at UC Davis for blood cell counts. Two control animals were injected with sterile saline on the same time course to monitor the effects of injection alone. Splenic aspiration (the dorsal and caudal aspect of the splenic parenchyma was aspirated via ultrasound guidance, 5 to 10 mL) was performed on all horses on days 0 and 35. Owing to problems associated with poor cell yield and preparation for flow cytometry analysis in day 0 samples, four additional healthy horses were recruited for splenic sampling, as described in the ‘Study design’ section.

### Serum cytokine analysis

Enzyme-linked immunosorbent assays for serum interleukin-6 (IL-6) (diluted 1:25; R&D Systems, Minneapolis, MN, USA), TGF-β1 (diluted 1:20, Human TGF-β1 Immunoassay; R&D Systems), and TNF-α (diluted 1:4, Equine TNF-α Screening Set; Thermo Scientific, Waltham, MA, USA) were performed exactly as previously described [3,18]. Serum for IFN-γ and IL-17 determination was submitted to the Animal Health Diagnostic Center, College of Veterinary Medicine, Cornell University (Ithaca, NY, USA).

### Flow cytometry

Mononuclear cells were isolated from blood and spleen by using gradient centrifugation (Ficoll; GE Healthcare, Pittsburg, PA, USA) in accordance with previously described protocols with the following modification [3]. Prior to gradient centrifugation, splenic samples were passed through a mesh strainer (Fisher Scientific, Pittsburgh, PA, USA) and brought to a final volume of 35 mL with sterile Dulbecco’s phosphate-buffered saline (DPBS) (Life Technologies, Frederick, MD, USA). For all labeling, a minimum of 10^5^ cells were centrifuged and resuspended in 100 μL of flow buffer: DPBS, 2% fetal bovine serum (Thermo Fisher, Waltham, MA, USA), and 1 mM EDTA (Sigma-Aldrich, St. Louis, MO, USA). The following antibodies were used: mouse-anti-equine CD3 (clone UC F6G 1:250; Jeffery Stott, University of California, Davis, CA, USA) [19], mouse-anti-human CD21 (clone B-ly4 1:20; BD Pharmingen, San Jose, CA, USA) [20,21], polyclonal goat-anti-human CD25 (clone AF-223; R&D Systems) [22], rat-anti-mouse/human FoxP3 (clone PCH101; ebioscience, San Diego, CA, USA) [22], mouse-anti-CD4 (clone HB61A 1:133; VMRD, Pullman, WA, USA) [22], mouse-anti-equine CD8 (clone F18P 1:500; J. Stott) [23], and a donkey-anti-mouse secondary when necessary (1:50; Jackson ImmunoResearch Laboratories, Inc., West Grove, PA, USA). All primary antibodies were incubated for 30 minutes and all secondary antibodies were incubated for 20 minutes in the dark. Cells were washed with 2 mL of flow buffer and centrifuged after each staining step and analyzed by flow cytometry as previously described [24]. All samples were run on a Cytomics FC500 flow cytometer (Beckman Coulter, Indianapolis, IN, USA) and analyzed with FlowJo flow cytometry software (Tree Star Inc., Ashland, OR, USA). The percentage of CD25^+^ cells and the percentage of FoxP3^+^ were gated from CD4^+^ cells.

### Statistical analysis

Data distribution was determined by using Shapiro-Wilk normality tests. All data were analyzed by using non-parametric analyses, and statistical significance was determined by using Kruskal-Wallis tests for bivariate data and Dunn’s multiple comparison tests for time course (multivariate) data (GraphPad Prism 6.02; GraphPad Software, Inc., La Jolla, CA, USA).

## Results

### Intravenous injections of allogeneic adipose tissue- or bone marrow-derived mesenchymal stem cells do not induce a systemic inflammatory response

**Figure 1:**
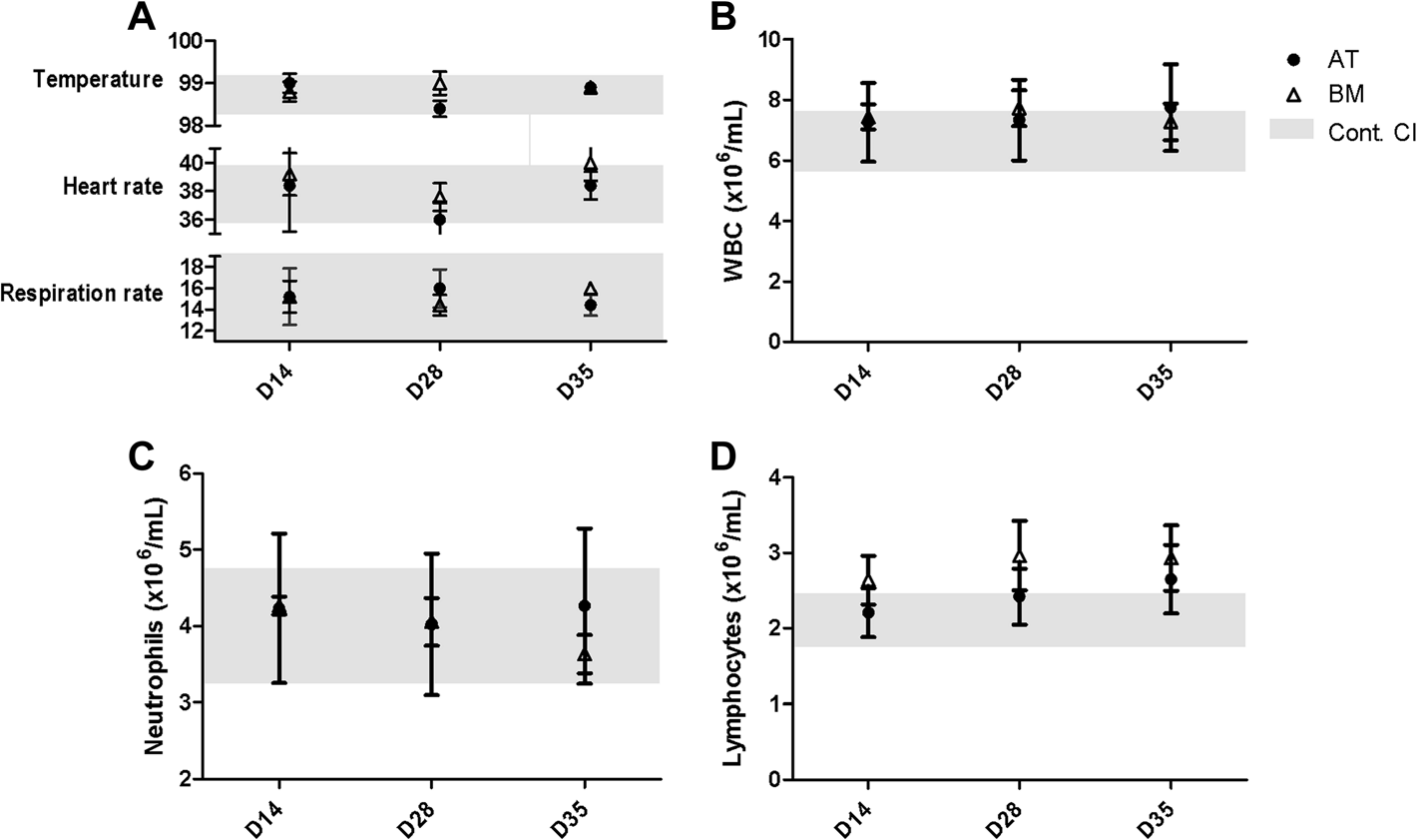
Multiple allogeneic mesenchymal stem cell (MSC) injections do not induce anaphylaxis or alter leukocyte concentration. **(A)** There were no significant changes in temperature, heart rate, or respiration rate throughout the course of MSC administration. **(B)** White blood cell count (WBC) did not change throughout the course of the study. Neutrophil **(C)** and lymphocyte **(D)** counts did not change throughout the course of the study. These data demonstrate that multiple high-dose injections of allogeneic MSCs do not result in an anaphylactic response and do not elicit a change in leukocyte concentration. Data are presented as mean ± standard error of the mean. Grey boxes demonstrate the 95% confidence interval (CI) of the day 0 and control samples. AT, adipose tissue; BM, bone marrow; D, day.

**Figure 2:**
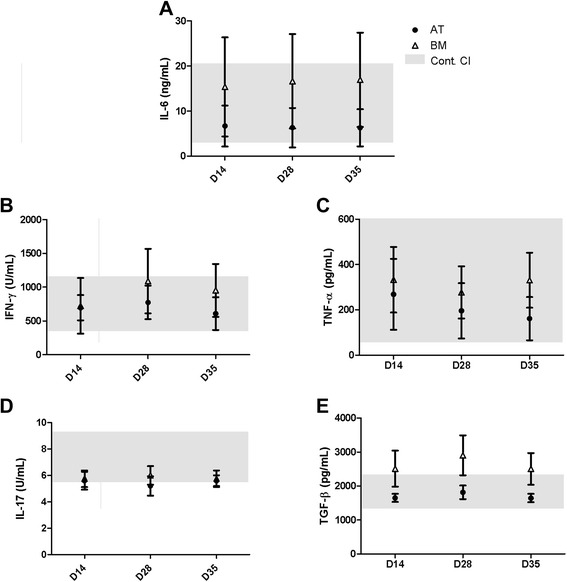
Multiple allogeneic mesenchymal stem cell (MSC) injections do not induce a systemic cytokine response. Serum concentration of pro-inflammatory cytokines **(A)** interleukin-6 (IL-6), **(B)** interferon-gamma (IFN-γ), and **(C)** tissue necrosis factor-alpha (TNF-α) did not change significantly as a result of multiple MSC injections. Neither **(D)** IL-17 nor **(E)** transforming growth factor-beta (TGF-β) was modulated by MSC administration. These data demonstrate that multiple high-dose injections of allogeneic MSCs do not result in a systemic inflammatory response. Data are presented as mean ± standard error of the mean. Grey boxes demonstrate the 95% confidence interval (CI) of the day 0 and control samples. AT, adipose tissue; BM, bone marrow; D, day.

Baseline data were collected on all horses prior to and after all MSC infusions. There were no significant changes in temperature, heart rate, or respiration rate as a result of MSC administration throughout the course of the study (Figure 1A). There were no significant changes in the absolute number of blood leukocytes, neutrophils, or lymphocytes over the course of the study (Figure 1B-D). There were also no significant changes in the serum concentration of IL-6, IFN-γ, IL-17, TGF-β, or TNF-α, further demonstrating that the MSCs did not elicit a systemic inflammatory response (Figure 2).

### Intravenous injections of allogeneic bone marrow-derived mesenchymal stem cell increase blood CD8^+^ T cells

**Figure 3:**
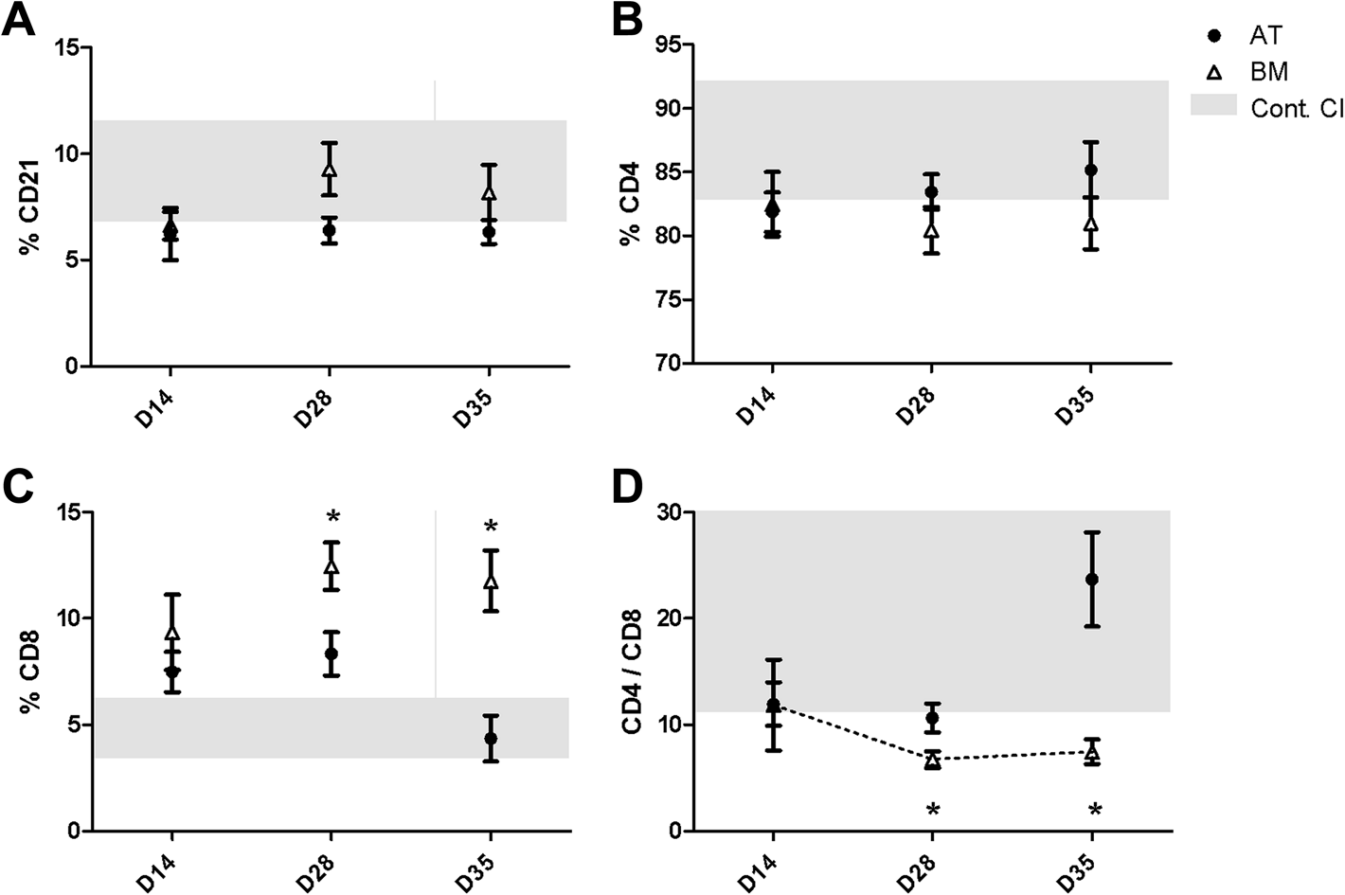
Multiple allogeneic mesenchymal stem cell (MSC) injections increase CD8^+^ T-cell numbers. There were no significant changes in the percentages of circulating CD21^+^ B cells **(A)** or CD4^+^ T cells **(B)** following multiple injections of MSCs. However, CD8^+^ T-cell percentages increased significantly in horses injected with bone marrow (BM)-derived MSCs (12.46% at day 28 and 11.76% at day 35) compared with control **(C)**. The increase in CD8^+^ T cells decreased the CD4/CD8 ratio significantly **(D)**. Data are presented as mean ± standard error of the mean. Grey boxes demonstrate the 95% confidence interval (CI) of the day 0 and control samples. *P <0.05. AT, adipose tissue; D, day.

We measured the percentage of circulating T- and B-cell numbers to determine whether multiple MSC injections altered circulating lymphocyte phenotype. The percentage of CD21^+^ B cells and CD4^+^ T cells did not change significantly in any of the experimental groups (Figure 3A, B). However, the percentage of CD8^+^ T cells was significantly increased on days 28 and 35 (12.5% ± 1.1% and 11.8% ± 1.4%, respectively) in horses injected with BM-MSCs compared with control horses (4.8% ± 0.7%). In contrast, the percentage of CD8^+^ T cells was not significantly elevated in horses that received AT-MSCs (Figure 3C). The changes in CD8^+^ T cells significantly decreased the CD4/CD8 T-cell ratio in horses that received BM-MSCs (6.73% ± 0.73% on day 28 and 7.45% ± 1.17% on day 35) compared with control horses (20.64% ± 4.82%, Figure 3D). These data demonstrate that repeated injections of BM-MSC elicit a greater CD8^+^ T-cell response when compared with controls but that AT-MSCs do not.

### Allogeneic intravenous injection of bone marrow-derived mesenchymal stem cells may increase splenic FoxP3 regulatory T cells

**Figure 4:**
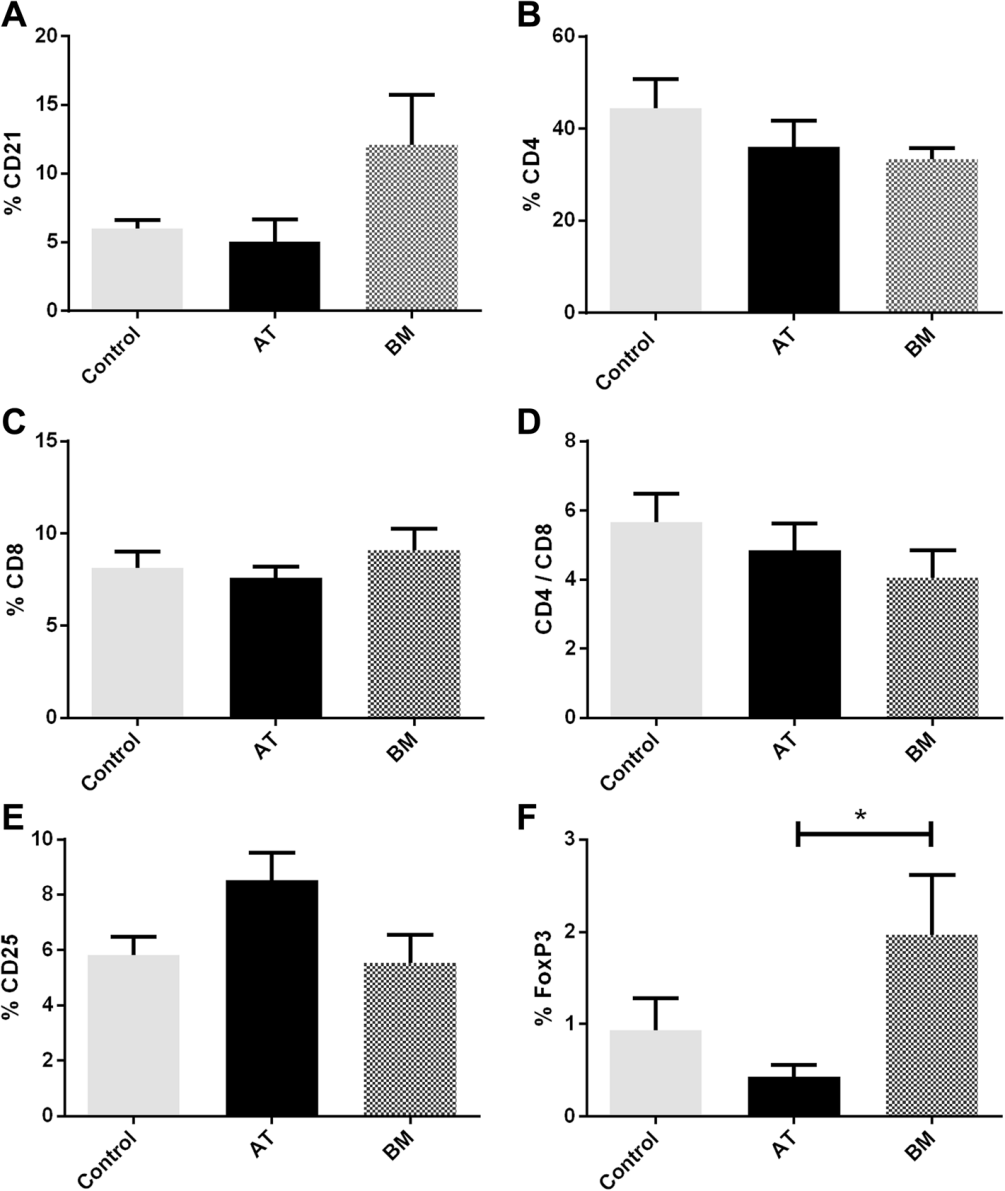
Multiple allogeneic mesenchymal stem cell (MSC) injections result in changes in splenic regulatory T cell percentages. **(A-D)** There were no significant changes in splenic CD21^+^ B-cell **(A)**, CD4^+^ T-cell **(B)**, or CD8^+^ T-cell percentages **(C)** or CD4/CD8 ratios **(D)** following multiple MSC injections. **(E)** There were no significant changes in activated (CD25^+^) lymphocyte proportions. **(F)** There were significantly higher percentages of splenic FoxP3^+^ regulatory T cells in the horses injected with bone marrow (BM)-derived MSCs compared with horses injected with adipose tissue (AT)-derived MSCs. Data are presented as mean ± standard error of the mean. *P <0.05.

Splenic lymphocyte subsets were evaluated one week after the final MSC injection in all horses. Three IV injections of allogeneic AT- or BM-MSCs did not change the percentage of splenic CD21^+^ B cells, CD4^+^ T cells, or CD8^+^ T cells (Figure 4A-C). Unlike the percentage of CD8^+^ T cells and the CD4/CD8 ratio in blood, the splenic lymphocyte CD4/CD8 ratio did not change after MSC injections (Figure 4D). These data demonstrate that whereas circulating numbers of CD8^+^ T cells are increased after repeated BM-MSC injections, splenic CD8^+^ T cells do not reflect or mimic this response to allogeneic MSCs regardless of the MSC source.

There were no significant changes in circulating FoxP3^+^ Treg cell numbers (data not shown). Interestingly, the number of splenic FoxP3^+^ Treg cells was significantly greater in horses that received BM-MSCs (2% ± 0.7%) compared with horses that received AT-MSCs (0.4% ± 0.1%) but not compared with control horses (0.9% ± 0.4%). (Figure 4E, F). These data show that whereas overall CD4^+^ T-cell numbers did not change as a result of MSC injection, Treg cell numbers may be influenced by MSC source.

## Discussion

Overall, the data demonstrate that multiple IV injections of allogeneic MSCs are well tolerated in horses. We administered three dosages of 25 × 10^6^ allogeneic MSCs derived from either AT or BM (a total of 75 × 10^6^ MSCs each), with two weeks between each injection. There were no measurable adverse clinical responses to infusion. We did observe increased circulating CD8^+^ T-cell numbers that suppressed the CD4/CD8 ratio in horses that received BM-MSCs. We also observed an elevated splenic FoxP3^+^ Treg cell population in horses that received BM-MSCs compared with horses that received AT-MSCs, but not compared with control. Although repeated IV injection of allogeneic MSCs from either AT or BM appears to be well tolerated in healthy horses, BM-MSCs might elicit an elevated CD8^+^ T-cell response and may also promote the development of Treg cells. Although no clinical signs were associated with these immune cell alterations, the underlying mechanism of these immune reactions and their potential longer-term clinical outcome should be addressed in future studies.

IV injection of MSCs is commonly used successfully in both human [25] and veterinary [1,26] clinical applications but is underutilized in equine medicine. Though not yet well studied in equine medicine, the IV injection route, in human patients, is highly advantageous for treatment of systemic inflammatory, infectious, or ischemic diseases in which local lesion injection is not feasible. MSCs are typically found in the lungs, gut, kidney, liver, thymus, and skin following IV transplantation in non-injury models and are further found at the site of injury in injured animal models [27]. A potential advantage of IV injection is that it may result in higher survival rates of MSCs following transplantation [28]. Additionally, systemic administration does not have the same injection volume limitations that can hinder local administration. As such, systemic IV MSC administration could potentially support larger cell doses than local administration.

Injections of 20 × 10^6^ MSCs have been previously administered to horses through local injection routes and are well tolerated [4]. More recently, a single IV injection of 0.2 to 1 × 10^6^ MSCs of allogeneic MSCs was shown to be well tolerated in clinically healthy horses. We chose a higher dose of MSCs for this study as the safety of multiple IV injections of allogeneic MSCs in the horse had not yet been documented. In human medicine, IV injection of 1 to 2 × 10^6^ MSCs per kilogram is a common dose for immunomodulatory applications of MSCs [25]. A comparative dose would be on the order of 10^8^ to 10^9^ MSCs in horses. We found that three serial injections of 25 × 10^6^ MSCs were well tolerated in healthy horses. However, a potential limitation of this study, based on published trials in human patients, is that a total of 75 × 10^6^ MSCs may not be therapeutically effective in horses for systemic treatment of immune-mediated diseases and disorders. If 10^8^ to 10^9^ cells are truly deemed necessary for efficacy in the treatment of equine systemic inflammatory diseases, cell bioreactors would be needed to generate these cell numbers.

There were no adverse reactions to AT-MSCs following IV injection. However, we did find increased CD8^+^ T-cell numbers following repeated injection of BM-MSCs in healthy horses. These data suggest that repeated BM-MSC injection may elicit a mild cytotoxic response to allogeneic antigens, which is consistent with findings in other healthy animal models [29]. Our laboratory has previously investigated the differences in the immunomodulatory properties of equine MSCs that are derived from different tissue sources, including AT and BM [3,15]. AT- and BM-MSCs were similar in their capacity to inhibit mitogen-induced T-cell proliferation and T-cell secretion of IFN-γ and TNF-α *in vitro* and were similar in their capacity to secrete the immunomodulatory mediators PGE_2_, TGF-β, and IL-6. However, BM-MSCs showed nitric oxide activity whereas AT-MSC did not [3]. Furthermore, the mechanism by which these MSCs decrease lymphocyte numbers *in vitro* also differs. AT-MSCs induced T-cell apoptosis in activated T cells, whereas BM-MSCs induced cell cycle arrest in similar conditions [15]. Nevertheless, none of these *in vitro* characteristics provides an explanation for the differences in CD8 T-cell numbers noted in the present study. It may be the low number of animals enrolled with unknown tissue matching. An additional consideration is that BM-MSCs are more heterogeneous, which may support CD8 T-cell activation at early passage, whereas AT-MSCs are more homogenous. Moreover, we have found that 37% of horses that received allogeneic MSC therapy (systemically and locally) developed MSC-specific antibody response (Sean Owens, in review). However, MSCs have also been shown to reduce immune response to allo-antigens in other species [17]. Importantly, allogeneic MSCs are being explored in human and veterinary clinical trials because they are readily available and they do not elicit acute or delayed immune-mediated hypersensitivity [1,10]. Finally, this study was limited to non-injury model animals. Previous studies have shown that MSC injection in non-injury model horses may elicit an inflammatory response [3,30]. Future applications of intravenously injected BM-MSCs should monitor for a CD8^+^ T-cell response to elucidate whether this change was a function of the non-injured model or a more consistent problem with repeated IV injection of allogeneic BM-MSCs in horses.

In human *in vitro* assays, MSCs inhibit T helper 17 (Th17) differentiation and activation and induce a Treg phenotype [31]. A previous study in horses has characterized phenotypic and functional properties of Treg cells [22]. Our findings in horses show that different MSC sources may uniquely impact splenic Treg cell numbers. Findings from our lab [15] and others [4,32] confirm that MSCs from different tissue sources vary in their functions. However, sampling limitations and the relatively non-uniform distribution of the cells that comprise the spleen hindered our ability to thoroughly investigate the ability of either MSC source to generate splenic Treg cells. Further testing in a disease model with a larger sample size is needed to demonstrate therapeutic efficacy of one MSC source over another and the impact that MSC source may have on Treg production *in vivo*.

## Conclusions

The results of this study demonstrate that repeated IV allogeneic MSC injections in horses are well tolerated. In light of the safety profile that was demonstrated in this study, further development of novel MSC therapies for equine orthopedic and immune-mediated diseases may be pursued.

## Abbreviations

AT: adipose tissue
AT-MSC: adipose tissue-derived mesenchymal stem cell
BM: bone marrow
BM-MSC: bone marrow-derived mesenchymal stem cell
DPBS: Dulbecco’s phosphate-buffered saline
IFNγ: interferon-gamma
IL: interleukin
IV: intravenous
MSC: mesenchymal stem cell
PGE_2_: prostaglandin E_2_
TGFβ: transforming growth factor-beta
Th: T helper
TNFα: tissue necrosis factor-alpha
Treg: regulatory T
